# Supplementary material for: Applied machine learning for the risk-stratification and clinical decision support of hospitalised patients with dengue in Vietnam
Source: PLOS Digit Health. 2022 Jan 18;1(1):e0000005. doi: 10.1371/journal.pdig.0000005 (PMC9931311; doi:10.1371/journal.pdig.0000005)
Supplement: S1 Appendix — (DOCX) [file pdig.0000005.s001.docx]

**Supplementary Appendix**

Appendix I – Description of data source and individual studies 2

Appendix II – Model development and hyperparameter tuning 8

Appendix III – Secondary and sensitivity analyses 13

**Supplementary appendix I**

**Description of data source**

Prospective clinical studies conducted in Vietnam by Oxford University Clinical Research Unit conducted between 12th April 2001 and 30th January 2018 were used to derive the final dataset used for analyses.

Electronic data from the following studies were accessed after a data sharing agreement between Imperial College London and Oxford University Clinical Research Unit (OUCRU)/ Hospital for Tropical Diseases (HTD) in March 2020. A summary of the included studies including the baseline characteristics of patients is shown in table A:

| No. | Study identifier code | Recruitment dates | Enrolled patients (n) | Title of study |
| --- | --- | --- | --- | --- |
| 1 | S1^1^ | 12/4/2001- 24/7/2009 | 3,042 | Inpatient-based study examining prognostic factors during the febrile phase |
| 2 | S2^2^ | 3/8/2009 – 8/12/2010 | 225 | Randomised control trial comparing use of steroids versus placebo in acute dengue |
| 3 | S3 | 08/12/2010 – 16/6/2011 | 88 | Prospective study in acute dengue |
| 4 | S4^3,4^ | 19/10/2010 – 4/12/2014 | 8,100 | Outpatient-based study examining prognostic factors during the febrile phase |
| 5 | S5 | 20/10/2016 – 30/1/2018 | 664 | Study of acute dengue in pregnancy during the febrile phase |

Table A – summary of included studies

**Data processing**

Studies were stored in an Excel format (Microsoft Corporation, Redmond, USA) and imported into Python 3.7 format using the pandas library ^5^. These were then concatenated into a single working dataset which consisted of data from 12,119 unique patient entries. Data was checked manually for inconsistencies and outliers and entries removed or cleaned. The initial data was in a “tidy” format where each row represents a unique patient/date combination.

Patients with a final diagnosis of dengue were determined according to the diagnostic criteria within the studies but consisted of one of: a positive NS1 point of care lateral flow assay, positive reverse transcriptase polymerase chain reaction (RT-PCR), positive dengue IgM through acute serology, or seroconversion of IgM. Patients without a confirmed diagnosis of dengue (n=6,329) were removed from the dataset.

Patients enrolled in the intervention arm of the randomised control trial S3 were then removed (n=150), as were patients who were not admitted to hospital and managed in the community – this consisted of the majority of patients in S4 which was an outpatient-based study.

For patients who experienced dengue shock syndrome we discarded all entries obtained on, and within the period when predictor variables were collected (up to 48 hours after admission). For patients who did not experience complication in dengue, we used a median illness day of 5 (i.e. up to 120 hours after onset of illness where start of illness is represented by day 0) as the cut-off and discarded all observations after that date (n=283). Laboratory values measured within the predictor period were summarised to minimum, median and maximum – for the majority of studies these investigations were performed daily. This formed the final dataset used for analysis (n=4,131 patients).

**Details of individual studies**

1. “Inpatient-based study examining prognostic factors during the febrile phase” (S1)

**Recruitment details**

This was a prospective inpatient observational study recruiting children between 5 and 15 years admitted to the paediatric dengue ward at the HTD, Ho Chi Minh City between 12/4/2001- 24/7/2009. A total of 3,042 patients were recruited to the study with confirmed dengue and 2,615 patients were included in the analyses after filtering.

Participants who are admitted to the paediatric ward at the HTD are those with uncomplicated illness only and not suitable for outpatient care. Patients with severe dengue or require frequent (3-6 hourly) monitoring are admitted or transferred to the paediatric intensive care unit.

**Dengue diagnosis**

Diagnosis of dengue is confirmed by detection of dengue virus (DENV) RNA in plasma by reverse transcriptase polymerase chain reaction (RT-PCR) at enrolment, or by seroconversion on IgM and IgG capture ELISA on paired enrolment and early convalescent specimens (Dengue Duo IgM and IgG Capture ELISA, PanBio, Australia) or in-house methods^6^.

**Outcomes**

The development of shock– the most common manifestation of severe dengue in this population was the primary endpoint. This was defined as narrow pulse pressure (≤ 20 mmHg) or hypotension for age with evidence of impaired peripheral perfusion ^7^.

1. “Randomised control trial comparing use of steroids versus placebo in acute dengue” (S2)

**Recruitment details**

This was a randomised placebo-controlled clinical trial examining the effects of low or high dose oral prednisolone in patients aged 5-20 years admitted to the HTD. In total, 225 participants were enrolled – these patients had a fever onset of ≤ 72 hours, did not present with signs of dengue-related complications, did not have past medical history or regular medications and had a confirmed diagnosis of dengue. Of this group, the 73 patients in the placebo group were included in the analyses.

Admission criteria for adults is based on HTD hospital guidelines but are in general consistent with warning signs defined by the World Health Organisation 2009 dengue guidelines^7^ suggestive of an elevated risk of progression to severe dengue. These include clinical signs such as persistent vomiting, abdominal pain or tenderness, skin or mucosal bleeding or signs of fluid leakage but also includes patients who have comorbidities or would benefit from closer clinical monitoring, at the discretion of the clinical team.

**Dengue diagnosis**

Diagnosis was supported through a positive rapid test for dengue nonstructural protein 1 (NS1 Ag-STRIP, Bio-Rad) and confirmed through either a DENV RT-PCR at enrolment or a capture ELISA IgM and IgM using paired specimens (Venture Technologies, Sarawak, Malaysia) within 72 hours of illness onset and at day 7.

**Outcomes**

Development of dengue shock syndrome according to WHO 2009^7^ dengue guidelines, and severe clinical bleeding as evaluated by clinician were used as outcome definitions.

1. “Prospective study in acute dengue” (S3) – unpublished data

**Recruitment details**

This was a prospective inpatient observational study recruiting patients aged 12-25 admitted to the HTD with early acute dengue (within 72 hours of illness onset) between 08/12/2010 – 16/6/2011. Admitted patients were followed up daily until discharge. In total 88 patients were enrolled of which 47 were included in the analyses after filtering. Admission criteria to the HTD is mentioned above.

**Dengue diagnosis**

Diagnosis of dengue is confirmed by detection of DENV-RNA in plasma by RT-PCR at enrolment or by IgM seroconversion using methods described above

**Outcomes**

Vascular leak and severe bleeding according to WHO 2009 dengue guidelines.

1. “Outpatient-based study examining prognostic factors during the febrile phase” (S4)

**Recruitment details**

This was a prospective community-based observational study recruiting patients presenting to outpatient departments at seven large hospitals in Southern Vietnam – these include the HTD, Children’s Hospital 1 and 2 in Ho Chi Minh city, Dong Nai children hospital, Long An provincial hospital, Tien Giang hospital, and Binh Duong hospital. Children between 1-15 years of age attending the outpatient with a fever 3 days or less in whom dengue is a possible diagnosis was eligible for enrolment. The exclusion criteria included cases where an alternative diagnosis is more likely or if the participant would be unlikely to be able to attend follow up. In total 8,100 participants were enrolled in the study of which 2,245 had confirmed dengue and 940 patients who were hospitalised were included in the analyses.

**Diagnosis**

A diagnosis of dengue was confirmed through one of the following: positive RT- PCR for DENV, a positive NS1 assay (Platelia NS1 antigen or NS1 Ag STRIP, Bio-Rad, France) at enrolment or detection of IgM seroconversion.

**Outcomes**

Dengue shock syndrome, dengue with severe bleeding, or dengue with end-organ involvement (central nervous dysfunction, hepatic dysfunction or severe respiratory dysfunction or other major organ involvement) constituted study endpoints.

1. “Study of acute dengue in pregnancy during the febrile phase” (S5) – unpublished data

**Recruitment details**

This was a prospective observational study recruiting women with acute dengue during pregnancy matched with non-pregnant women controls with acute dengue at the HTD in Ho Chi Minh City. Women between 18 and 45 years old referred to the HTD with a fever and clinical symptoms consistent with dengue and who were either pregnant or not during the recruitment period were eligible.. The matching criteria were patients’ age and day of illness at enrolment. In total 664 patients with dengue were recruited of which 456 were included in the analyses after filtering.

**Diagnosis**

Diagnosis of dengue is confirmed by detection of DENV-RNA in plasma by RT-PCR, NS1 antigen detection at enrolment or by IgM seroconversion using methods described above

**Outcomes**

Patient outcomes are classified according to WHO 2009 dengue guidelines which include development of DSS, significant clinical bleeding or organ involvement.

**Supplementary appendix II**

**Description of statistical methods and results**

Work was conducted using the NumPy^8^, pandas^5^ and Scikit-learn^9^ libraries in Python 3.7.

From the final dataset described in appendix I, we transformed the data through grouping by patient and date in order to derive observations corresponding to the first 48 hours of hospital admission for each patient. The minimum, maximum and median values for laboratory tests for haematocrit and platelet counts were derived from this 48 hour period and used in the final “tidy” data format.

**Feature selection**

Feature selection was pragmatic and we selected those indices which would be available across a diverse set of healthcare settings. Haematocrit^7^ and platelet^10^ change over time are valid prognostic markers during the febrile phase reflecting plasma leak and disease severity respectively and were therefore chosen as predictors. Although other laboratory variables including serotype, viral load, liver and biochemical profiles were available and are prognostic indicators in dengue, and certain machine learning algorithms (such as XGBoost and other ensemble-based approaches) are able to take this into account, the significant proportion of missing data (>10%) would affect ultimately affect robustness of the final model performance and were therefore not chosen.

The final predictors chosen were as follows:

1. Age in years
2. Sex (Male/Female)
3. Weight in kilograms
4. Day of illness on enrolment
5. Haematocrit in %
6. Platelet count × 10^9^ cells/L

Missing data is shown graphically in figure A and consists of <1.5% missing values for each variable, the distribution assumed to be missing at completely random.


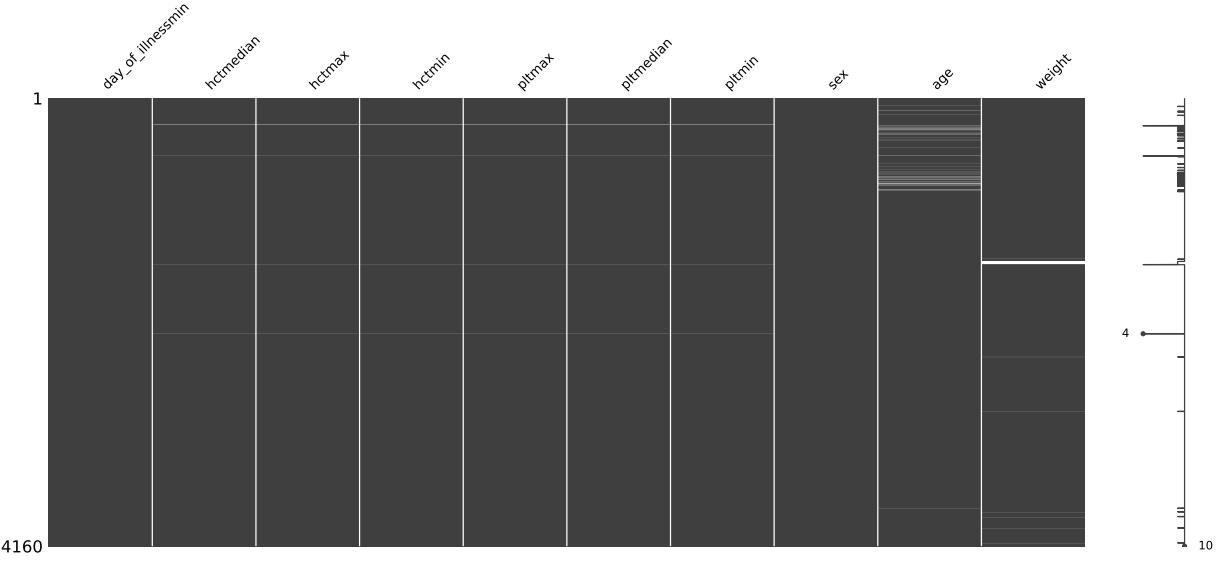


**Figure A. Data for 4,131 patients with columns indicating variables white lines representing missing data.**

We examined the selected features for collinearity through Pearson correlation grid, which is shown as follows in figure B:


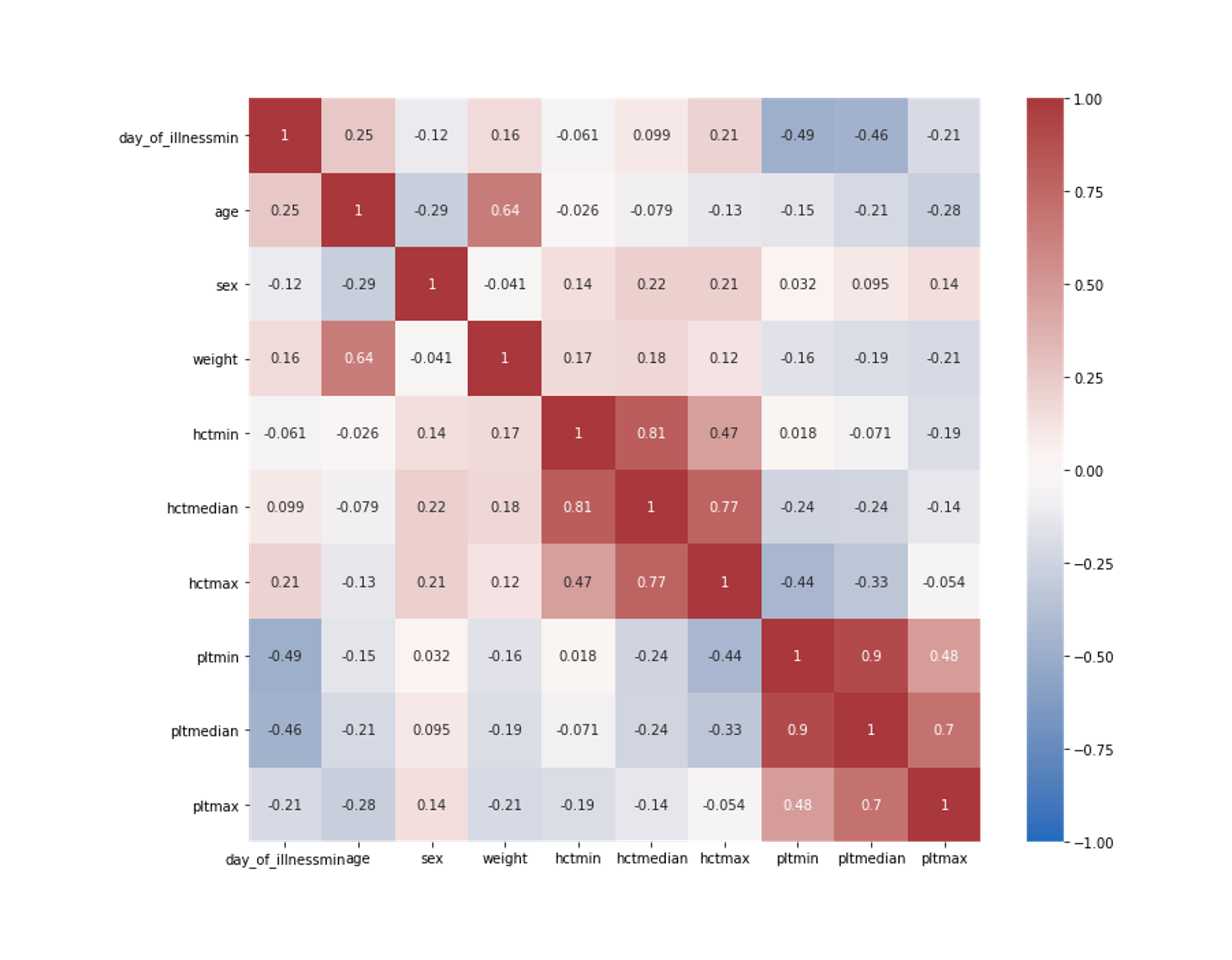


**Figure B. Pearson correlation grid for features included in the final models to examine for collinearity. Minimum, median platelets and haematocrit were correlated with a coefficient of 0.9 and 0.81 respectively but other features were generally independent from each other.**

**Data splitting, pre-processing and imputation**

The data was split into a train and test set at 80:20 ratio respectively through a stratified approach to ensure even distribution of outcomes in both development and hold-out test set.

Univariate comparison between the development and hold-out test set is as follows in table B:

|  | Development set (3,304) | Hold-out test set (827) | p-value |
| --- | --- | --- | --- |
|  | Median | Median |  |
| Shock (%) | 178 (5.4%) | 44 (5.3%) |  |
| Median age (years) | 12 (9-14) | 12 (10-14) | 0.06 |
| Median weight (kg) | 35 (27-45) | 36 (28-45) | 0.2 |
| Female sex (%) | 1615 (49%) | 393 (48%) | 0.51 |
| Median haematocrit (%,) | 39.6 (37.45-42) | 39.8 (37.7-42.29) | 0.13 |
| Maximum haematocrit (%) | 42 (39-45.6) | 42.25 (39.23-45.6) | 0.20 |
| Minimum haematocrit (%) | 37.6 (35.3-40) | 37.9 (35.4-40) | 0.11 |
| Median platelet count (x 10^9^/L) | 114.5 (80-156.5) | 111 (79.93-159) | 0.24 |
| Maximum platelet count (x 10^9^/L) | 158 (117.5-207) | 156 (116.25-203) | 0.17 |
| Minimum platelet count (x 10^9^/L) | 84 (49-133) | 81.35 (46.85-131) | 0.23 |

**Table B. Univariate analyses between development and hold-out test set after random stratified splitting, comparisons made by Mann-Whitney testing.**

Missing data was then imputed after fitting on the development set and applied to both the development and hold-out test set in order to prevent data leakage. The following method was used: categorical variables (namely missing sex) were imputed using the mode and numerical variables would either be imputed using the mean or median, then either undergo transformation by standardisation (variables scaled to unit variance) or not. SMOTE was used to account for class imbalance across all datasets and carried forward in the final model depending on the AUROC achieved in cross-validation. This pipeline was then fed into the grid search for individual hyperparameter tuning.

**Model development and validation**

Five models were chosen for evaluation, these include XGboost^11^ , random forest classifier^12^, logistic regression, k-nearest neighbour^13^, artificial neural networks^14^ and support vector machines^15^.

A repeat stratified 10-fold cross validation strategy, repeated 3 times (i.e., 30 train/test sets) was adopted, and hyperparameter tuning done through grid search.

Each model from each algorithm with a particular hyperparameter combination was tested through the cross-validation process with the area under the receiver operator curve (AUROC) as the primary scoring method. An isotonic calibration was subsequently applied to the models^16^.

The final models and their respective hyperparameter variables trained on the development and their hyperparameters are as follows:

1. **Extreme gradient boosting (XGBoost)**

eta': [0.005,0.01,0.05],

'min_child_weight': [0.005,0.01,0.05],

'max_depth': [3,4,5],

'gamma': [0.1,0.2,0.25],

'n_estimators':[50,100,250]

And

'eta': [0.05,0.1,0.2],

'min_child_weight': [0.05,0.1,0.2],

'max_depth': [5,7,10],

'gamma': [0.05,0.1,0.2],

'n_estimators':[25,100,200],

'subsample': [0.25,0.5,1],

'cosample_by_tree': [0.5, 0.7, 1],

'alpha': [0, 0.2, 0.5]

Optimal parameters: XGBClassifier(eta=0.01,gamma=0.1, max_depth= 4,min_child_weight=0.005, n_estimators= 250, eval_metric='logloss') with missing values handled by the algorithm.

1. **Random forest classifier:**

'criterion': ['gini','entropy'],

'n_estimators': [250, 500, 1000, 1500, 2000],

'min_samples_split': [8, 12, 14, 16],

'max_depth': [5, 8, 10, 20, 40, None]

Optimal parameters: RandomForestClassifier(criterion='entropy', max_depth=8, min_samples_split=8, n_estimators=500 with mean imputation and no scaling.

1. **Logistic regression**

‘solver': ['liblinear'],

‘penalty': ['LASSO', 'Ridge regularisation'],

'C': [0.01, 0.1, 1, 10, 100]

Optimal parameters: LogisticRegression(C=10, penalty='LASSO', random_state=42, solver='liblinear') with median imputation and no scaling.

1. **Artificial neural networks**

‘solver': ['sgd','adam'],

‘activation': ['relu'],

'hidden_layer_sizes': [(100,),(150,),(200,100)],

'alpha': [0.0001,0.001,0.05],

'max_iter': [200,400,600]

Optimal parameters: MLPClassifier(alpha=0.1, hidden_layer_sizes=(100, 100), max_iter=50)) with median imputation and standard scaling of numerical feature variables.

1. **Support vector machines**

kernel: [rbf],

‘C':[0.001,0.01,0.1],

'gamma':[0.01,0.1,1],

'probability':[True]

Optimal parameters: SVC(C=0.1, gamma=0.1, probability=True) with median imputation, standard scaling and SMOTE(sampling_strategy=0.1).

As a probabilistic classification was given, the optimal cut-off was determined using the maximum J-statistic, in order to maximise sensitivity and specificity and the default cut off of >0.50 not implemented

The performances of the models for each of the algorithms from cross validation of the development set as well as against the hold-out test set is shown in the main text.

**Bootstrapping**

In order to derive the confidence intervals for the performance metrics including AUROC, sensitivity, specificity, negative predictive value and positive predictive value, we performed percentile bootstrapping^17^ of the development set in order to generate, 1,000 different datasets.

Data were resampled with replacement at random through the bootstrapping procedure and underwent repeated testing with 90% of the bootstrapped dataset used to train the final model, and tested against the remaining 10% from the out of bag sample.

The distribution of each of the performance metrics was plotted and assumed to be gaussian in distribution, and the 95% interval was taken as the cut offs values at the 2.5 and 97.5 percentile of these values as 1.96 of the standard deviation from the mean.

**Interpretability and SHAP analysis**

A Shapley Additive Explanations analysis using the SHAP package^18^ was performed to understand the underlying relationships between the predictors and the data. The best performing model (XGBoost) and its performance on the hold out test set was fitted to the TreeExplainer function – the summary of SHAP values for each predictor is shown in figure C, and a scatter graph of predictor values with SHAP values for the XGBoost and logistic regression shown in figure D.


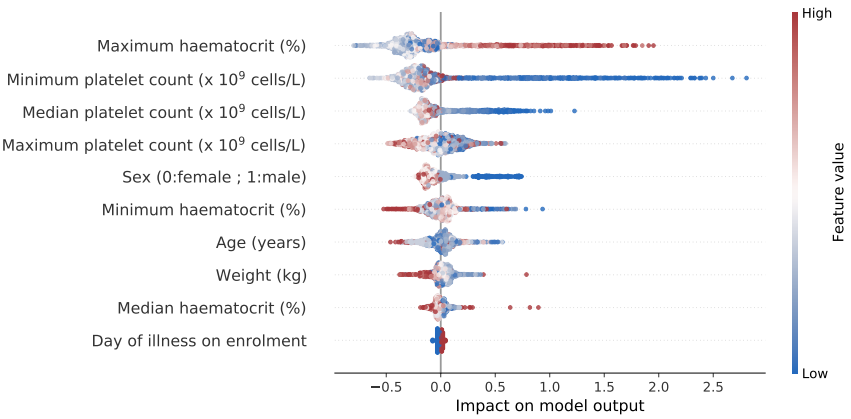


**Figure C. Summary plot for the variables used in the model in descending order of importance. Dengue outcomes are encoded as 1: complicated, 0: non-complicated. The colour gradients represent the value of the predictor – for example a lower minimum platelet count was associated with a positive impact on the model output in predicting a complicated dengue outcome.**


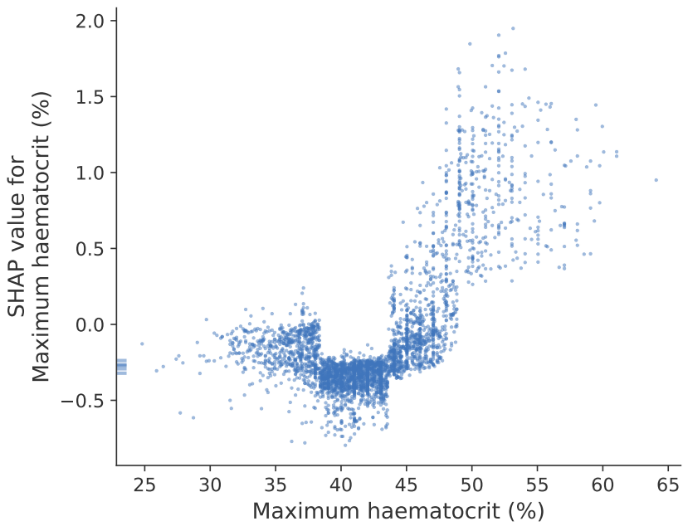

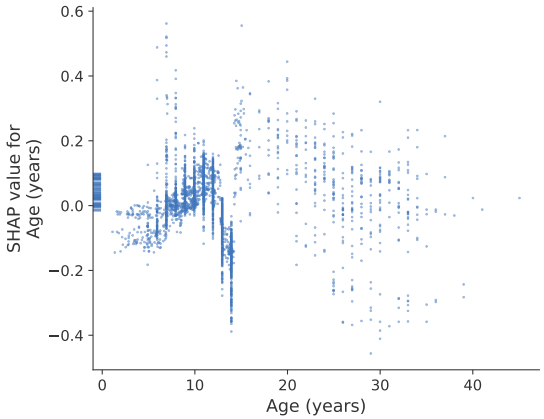

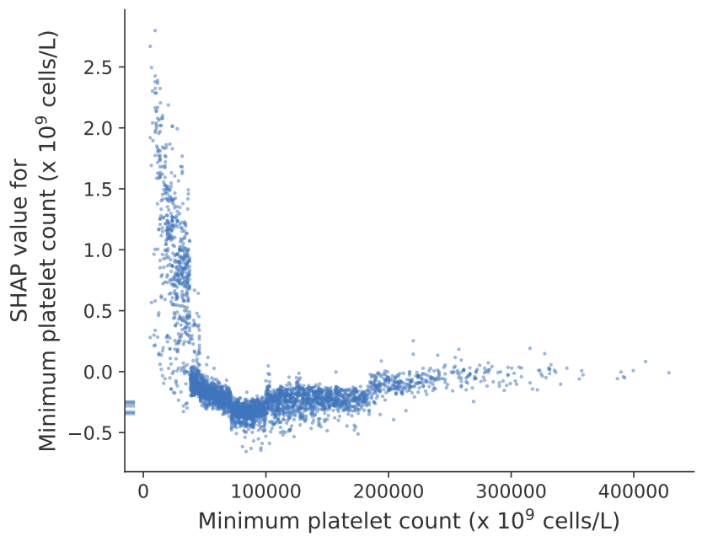

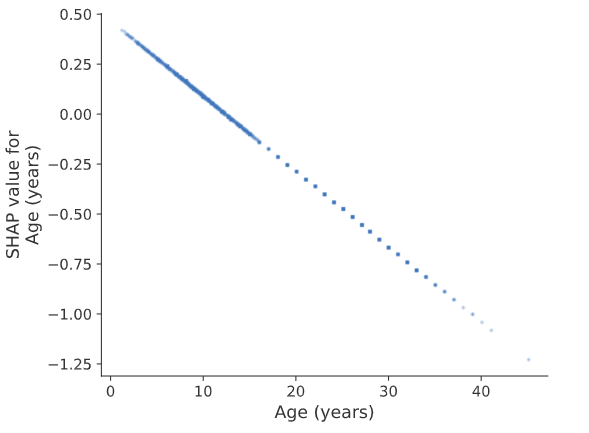

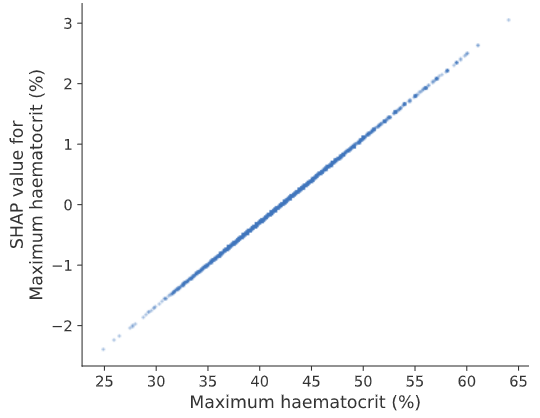

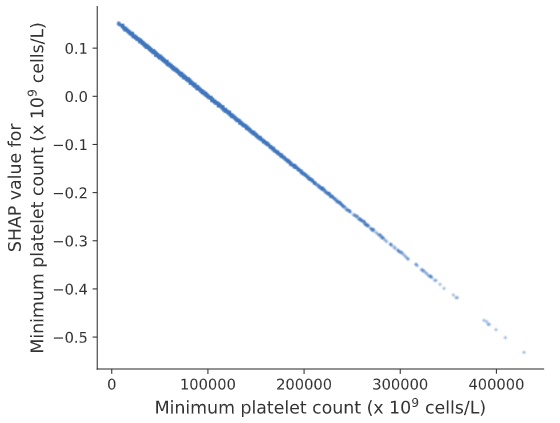


**Figure D. Scatter plots of transformed predictor values (x axis) and SHAP values (y axis). Outputs from the XGBoost is displayed on the left column, and logistic regression on the right. SHAP values are an indicator of feature importance with regards to the model output for the model when tested against the hold-out set.**

SHAP analyses were conducted for the other optimised models (ANN, LR, RFC and SVC) using the KernelExplainer function, using trained models tested on a smaller subset within the hold out test set (n=400). The results show that minimum platelet count and maximum haematocrit were consistently most important features, shown in figure E:


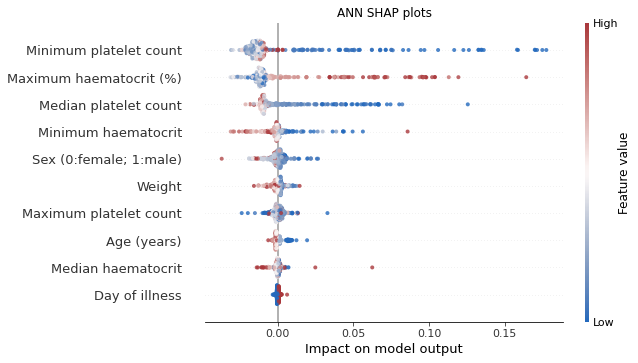

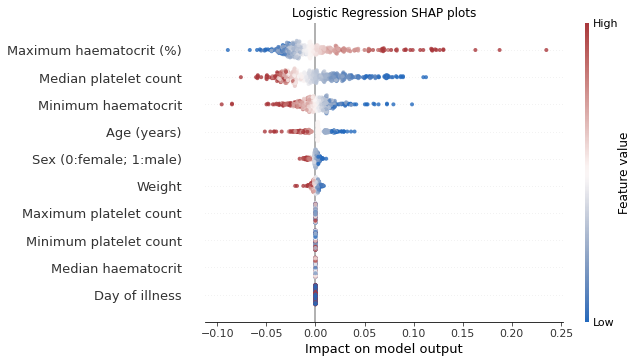

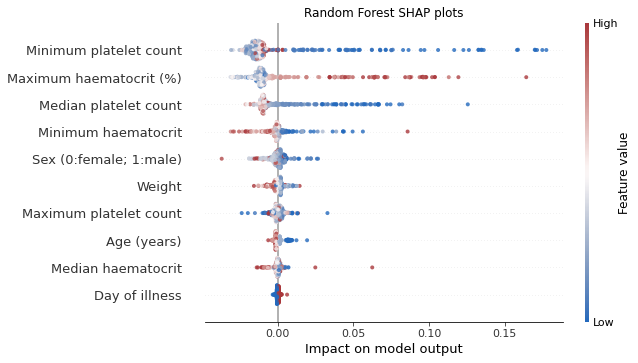

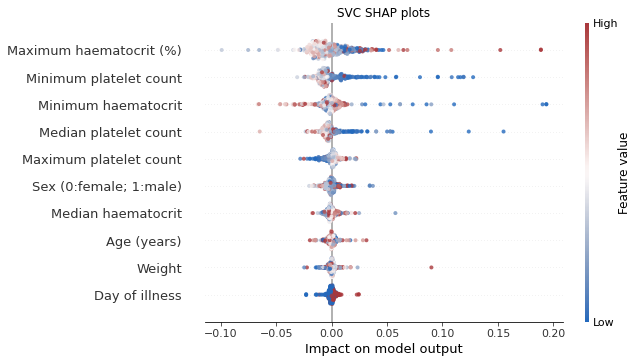


**Figure E. Scatter plots of transformed predictor values (x axis) and SHAP values (y axis) across other models.**

**Supplementary appendix III**

**Secondary and sensitivity analyses**

1. **Complicated dengue as an endpoint**

We developed machine learning and linear logistic regression models based on broader composite outcomes of “complicated dengue”. This classification encompasses features consistent with moderate and severe dengue which are clinically-relevant for hospital risk stratification of patients. We defined complicated dengue as development of any of the following features during hospitalisation:

- Dengue shock syndrome (DSS): rapid and weak pulse and narrow pulse pressure (≤ 20 mmHg) or hypotension for age, and clinical signs of hypotension.
- Clinically significant plasma leakage defined as clinically apparent fluid overload, requirement for ventilation, oxygenation or diuresis
- Significant bleeding including haematemesis, melaena but excluding skin and minor mucosal bleeding.

However the limitations of the data were such that apart from DSS, times of leakage or bleeding onset were not captured. Although the features variables are reflective of dengue in the early phase, the final models are taken to show *associations* with complicated dengue rather than serve as a predictor model.

Specific outcomes were extracted from each of the studies and shown in figure C:

| Study | Category | Data availability |
| --- | --- | --- |
| S1 | Shock | DSS |
|  | Leakage | Clinical fluid overload |
|  | Bleeding | Gastrointestinal bleeding |
| S2 | Shock | DSS |
|  | Leakage | Not available |
|  | Bleeding | Not available |
| S3 | Shock | DSS |
|  | Leakage | Treatment with invasive ventilation, oxygen, diuretics, presence of ascites |
|  | Bleeding | Severe mucosal bleeding, gastrointestinal bleeding |
| S4 | Shock | DSS |
|  | Leakage | Clinical fluid overload, treatment with oxygen, diuretics |
|  | Bleeding | Severe bleeding assessed by a clinician |
| S5 | Shock | DSS |
|  | Leakage | Severe leakage assessed by a clinician |
|  | Bleeding | Respiratory distress, ascites, pulmonary oedema |
| S6 | Shock | DSS |
|  | Leakage | Respiratory distress, ascites |
|  | Bleeding | Gastrointestinal bleeding |

**Table C – clinical endpoints extracted from individual studies**

**Methods**

The statistical analysis workflow followed that of the primary analysis. In brief the same set of feature variables as with the primary model was used (age, sex, weight, day of illness onset on enrolment, and indices of haematocrit and platelets over the first 48 hours of hospital admission). Machine learning models including XGBoost, random forest, artificial neural network, support vector machines and linear logistic regression were utilised. Data splitting into a development and hold out set was done randomly at a 80/20 ratio stratified according to complications. Optimisation was done through 10-fold cross validation on the development set only with area under the receiver operating curve (AUROC) used as the primary scoring method. See main text and supplementary appendix II for full details of methodology.

**Results**

In total, 4,131 patients were included for analysis – this is the same cohort as that used in the primary analysis. In total 344 (8.3%) patients experienced the composite complicated dengue endpoint during their hospitalisation.

A baseline description of patients included is shown in table D:

|  | complication n = 344 | no complication n = 3858 | p-value | Missing data (%) |
| --- | --- | --- | --- | --- |
|  | Median (IQR) | Median (IQR) |  |  |
| Median age (years ) | *11 (9-13)* | 12 (9-14) | <0.001 | 1.5 |
| Median day of illness at hospital admission (days) | *3 (2-4)* | 3 (2-4) | 0.02 | 0 |
| Median weight (kg) | *34 (26-43)* | 36 (27-45) | 0.03 | 0.9 |
| Female sex (%) | *157/344 (46%)* | 1890/ 3858 (49%) | 0.3 | 0 |
| Median haematocrit (%) | *41 (38-43)* | 40 (37-42) | <0.001 | 0.2 |
| Maximum haematocrit (%) | *45 (40-49)* | 42 (39-45) | <0.001 | 0.2 |
| Minimum haematocrit (%) | *38 (35-40)* | 38 (35-40) | 0.1 | 0.2 |
| Maximum platelet count (x 10^9^/L) | *136 (103-175)* | 158 (118-208) | <0.001 | 0.2 |
| Median platelet count (x 10^9^/L) | *85 (52-128)* | 116 (83-160) | <0.001 | 0.2 |
| Minimum platelet count (x 10^9^/L) | *50 (23-122)* | 86 (51-135) | <0.001 | 0.2 |

**Table D. Baseline description of patients (n=4,131) included in analysis. Haematological values and ranges refer to results taken over the initial 48 hours of hospitalisation only. Data is presented as median and brackets denote the interquartile range. Univariate analyses were done using the Mann-Whitney test.**

The performance of models developed using the development set using 10-fold cross validation are shown in table E. Performances were commensurate across all algorithms – similar to primary analysis the XGBoost algorithm showed reasonable discrimination with a mean AUROC of 0.759 (95% CI 0.731-0.805).

| Model | Mean AUROC | Specificity | Sensitivity | Positive Predictive value | Negative Predictive value | Brier score |
| --- | --- | --- | --- | --- | --- | --- |
| XGBoost | 0.759 (0.731-0.805) | 0.75 (0.632-0.873) | 0.668 (0.54-0.782) | 0.198 (0.167-0.308) | 0.961 (0.946-0.97) | 0.068 |
| Random forest | 0.766 (0.712-0.787) | 0.668 (0.606-0.857) | 0.745 (0.523-0.789) | 0.172 (0.144-0.266) | 0.966 (0.947-0.972) | 0.068 |
| Logistic regression | 0.701 (0.663-0.74) | 0.627 (0.485-0.838) | 0.689 (0.445-0.815) | 0.145 (0.119-0.208) | 0.956 (0.94-0.97) | 0.075 |
| Artificial neural networks | 0.758 (0.714-0.791) | 0.744 (0.592-0.856) | 0.65 (0.512-0.805) | 0.19 (0.143-0.259) | 0.958 (0.948-0.973) | 0.068 |
| Support vector machines | 0.66 (0.748-0.848) | 0.654 (0.537-0.854) | 0.45 (0.775-0.782) | 0.15 (0.127-0.233) | 0.954 (0.939-0.966) | 0.070 |

**Table E. Performance of models for each algorithm with respect to internal 10-fold cross validation on the development set (n=3,304). The 95% confidence intervals shown in brackets were derived from bootstrapping of the development set to 90% of original size and tested against the out of bag samples.**

Evaluation of calibrated models against the hold out validation (n=827) set showed that both artificial neural network and random forest classifier had best discrimination (AUROC 0.771) with a negative predictive value of 0.974 and 0.968 respectively (Table F).

| Model | Mean AUROC | Specificity | Sensitivity | Positive Predictive value | Negative Predictive value | Brier score |
| --- | --- | --- | --- | --- | --- | --- |
| XGBoost | 0.773 | 0.775 | 0.681 | 0.220 | 0.963 | 0.072 |
| Random forest | 0.782 | 0.694 | 0.750 | 0.186 | 0.968 | 0.069 |
| Logistic regression | 0.714 | 0.726 | 0.583 | 0.165 | 0.949 | 0.078 |
| Artificial neural networks | 0.782 | 0.582 | 0.833 | 0.156 | 0.974 | 0.070 |
| Support vector machines | 0.738 | 0.751 | 0.611 | 0.186 | 0.954 | 0.071 |
|  |  |  |  |  |  |  |

**Table F. Performance metrics of final models when tested against the hold-out set (n=847).**

**Summary**

Machine learning models using the less specific and broader classification of “complicated dengue” as an outcome showed reduced discrimination in terms of AUROC when trained and evaluated the aggregated patient cohort.

The limitations of the dataset have meant that the models describe only associations of feature variables with the outcome of complicated dengue and do not serve as predictive models. Nonetheless the characteristics of the model which show a high negative predictive value may still be of clinical utility for a decision support tool whereby patients at a low risk of experiencing features consistent with complicated disease can be automatically identified during their early hospital admission – this could be of benefit in informing novel strategies in organising clinical care especially in our setting. We plan to incorporate this model for prospective testing alongside the primary model which focuses on DSS.

1. **Exploring batch effects through dataset stratification**

We examined the contribution of batch effects of each individual dataset through a leave one group out analysis. Each study (S1 – S5) was used in turn for testing in 10-fold cross-validation for all models through a new grid search process to ensure that models were optimised.

Following results highlight heterogeneity between datasets: we found performance varied significantly between batches. One limitation is that the study size varies and we found that performance was worse overall when smaller samples were used for model training.

| Study used for testing in CV | AUROC |
| --- | --- |
| S1 (n= 2,615) | 0.50 |
| S2 (n= 73) | 0.94 |
| S3 (n=47) | 0.78 |
| S4 (n=940) | 0.46 |
| S5 (n=456) | 0.83 |

**Table G. Performance of XGBoost model in leave one group out cross validation**

|  | CV AUROC results (IQR) |
| --- | --- |
| XGB | 0.78 (0.50-0.83) |
| RFC | 0.84 (0.50-0.84) |
| LR | 0.84 (0.80-0.90) |
| ANN | 0.78 (0.46-0.85) |
| SVC | 0.73 (0.44-0.90) |

**Table H. Summarised results for all models in leave one group out cross validation**

We proceeded to stratify the dataset by the WHO classification used (1997 vs 2009. The outcome definitions of dengue shock are similar between the two but DHF I and II classifications in the former have been superseded by dengue with warning signs in the later classification schemes. Optimised models were evaluated using 10-fold cross validation using either studies which utilised the 1997 classification (S1, n=2,615) or the 2009 classification (S2-S5, n= 1,516).

We found that models applied to S1 showed better performance overall compared with those applied to S2-S5 – again limitations include the relative size of studies, and S2-S5 were overall much more heterogenous in nature and this would account for the difference in performances.

|  | 1997 classification | 2009 classification |
| --- | --- | --- |
|  | AUROC in 10-fold CV | AUROC in 10-fold CV |
| XGB | 0.61 | 0.87 |
| LR | 0.69 | 0.88 |
| RFC | 0.62 | 0.87 |
| ANN | 0.58 | 0.87 |
| SVC | 0.61 | 0.88 |

**Table I. Model performances in 10-fold cross valuation between studies using 1997 and 2009 WHO dengue treatment guideline classification.**

Each patient included in the clinical studies S1-S5 are similar and comparable to each other, insofar as they are all admitted to hospital with a confirmed diagnosis of dengue, clinical data collected during the febrile phase within 48 hours of admission and these data are collected prior to development of dengue shock.

However important differences between each study–patients recruited may also be subjected to different inclusion criteria and clinical triage processes, access to healthcare may be also be different depending on study location and timing. Furthermore, seasonality during recruitment periods will influence the dengue caseload, with studies conducted during seasonal epidemics or during circulation of particular virus serotypes resulting in more severe disease seen.

Although methods exist for batch correction such as individual dataset normalisation these have been mainly applied to -omics analysis and may be poorly suited to this study. Furthermore as each study is narrow in its focus, its concatenation would allow for improved prospective generalisability when applied to a real-world clinical cohort.

1. **Performance stratified by age**

We examined the performance of models when the dataset was stratified by age i.e. between adults (n=499) and children (n=3,571).

We developed models trained on using the entire paediatric cohort (defined as age <18) without splitting using 10-fold cross validation.

The results are as follows:

|  | ROC | Spec | Sens | PPV | NPV |
| --- | --- | --- | --- | --- | --- |
| XGB | 0.82 | 0.70 | 0.75 | 0.13 | 0.98 |
| LR | 0.79 | 0.70 | 0.75 | 0.13 | 0.98 |
| RFC | 0.82 | 0.90 | 0.56 | 0.27 | 0.97 |
| ANN | 0.82 | 0.82 | 0.66 | 0.18 | 0.98 |
| SVC | 0.83 | 0.82 | 0.67 | 0.19 | 0.98 |

**Table J. Optimal performance metrics of different models in 10-fold cross validation using the paediatric subset only (n=3,571)**

We then evaluated performance of these models when applied to adult patients (n=499, with 13 cases of dengue shock) as a test set:

|  | ROC | Spec | Sens | PPV | NPV |
| --- | --- | --- | --- | --- | --- |
| XGB | 0.70 | 0.90 | 0.47 | 0.12 | 0.98 |
| LR | 0.66 | 0.97 | 0.33 | 0.24 | 0.98 |
| RFC | 0.71 | 0.98 | 0.33 | 0.31 | 0.98 |
| ANN | 0.65 | 0.91 | 0.40 | 0.12 | 0.98 |
| SVC | 0.64 | 0.98 | 0.27 | 0.29 | 0.98 |

**Table K. Test performance of the models trained on the paediatric cohort and applied to the adult set.**

Overall performance was worse when applied to the adult patients however limitations in this analysis include the major class imbalance in the adult test set. The clinical management of dengue in paediatric and adult populations is different and future models need to balance the generalisability of a unified model with that which is optimised for specific age groups.

References

1 Lam PK, Ngoc TV, Thu Thuy TT, *et al.* The value of daily platelet counts for predicting dengue shock syndrome: Results from a prospective observational study of 2301 Vietnamese children with dengue. *PLoS Negl Trop Dis* 2017; **11**. DOI:10.1371/journal.pntd.0005498.

2 Tam DTH, Ngoc TV, Tien NTH, *et al.* Effects of Short-Course Oral Corticosteroid Therapy in Early Dengue Infection in Vietnamese Patients: A Randomized, Placebo-Controlled Trial. *Clin Infect Dis Off Publ Infect Dis Soc Am* 2012; **55**: 1216–24.

3 Nguyen MT, Ho TN, Nguyen VVC, *et al.* An Evidence-Based Algorithm for Early Prognosis of Severe Dengue in the Outpatient Setting. *Clin Infect Dis Off Publ Infect Dis Soc Am* 2017; **64**: 656–63.

4 Tuan NM, Nhan HT, Chau NVV, *et al.* Sensitivity and Specificity of a Novel Classifier for the Early Diagnosis of Dengue. *PLoS Negl Trop Dis* 2015; **9**. DOI:10.1371/journal.pntd.0003638.

5 McKinney W. Data Structures for Statistical Computing in Python. Austin, Texas, 2010: 56–61.

6 Shu P-Y, Chang S-F, Kuo Y-C, *et al.* Development of group- and serotype-specific one-step SYBR green I-based real-time reverse transcription-PCR assay for dengue virus. *J Clin Microbiol* 2003; **41**: 2408–16.

7 Special Programme for Research and Training in Tropical Diseases, World Health Organization, editors. Dengue: guidelines for diagnosis, treatment, prevention, and control, New ed. Geneva: TDR : World Health Organization, 2009.

8 Harris CR, Millman KJ, van der Walt SJ, *et al.* Array programming with NumPy. *Nature* 2020; **585**: 357–62.

9 Pedregosa F, Varoquaux G, Gramfort A, *et al.* Scikit-learn: Machine Learning in Python. *J Mach Learn Res* 2011; **12**: 2825–30.

10 Lam PK, Ngoc TV, Thu Thuy TT, *et al.* The value of daily platelet counts for predicting dengue shock syndrome: Results from a prospective observational study of 2301 Vietnamese children with dengue. *PLoS Negl Trop Dis* 2017; **11**: e0005498.

11 Chen T, Guestrin C. XGBoost: A Scalable Tree Boosting System. *Proc 22nd ACM SIGKDD Int Conf Knowl Discov Data Min* 2016; : 785–94.

12 Breiman L. Random Forests. *Mach Learn* 2001; **45**: 5–32.

13 Fix E, Hodges JL. Discriminatory Analysis. Nonparametric Discrimination: Consistency Properties. *Int Stat Rev Rev Int Stat* 1989; **57**: 238–47.

14 Hinton GE. Connectionist learning procedures. *Artif Intell* 1989; **40**: 185–234.

15 Platt JC. Probabilistic Outputs for Support Vector Machines and Comparisons to Regularized Likelihood Methods. In: Advances in Large Margin Classifiers. MIT Press, 1999: 61–74.

16 Niculescu-Mizil A, Caruana R. Predicting good probabilities with supervised learning. In: Proceedings of the 22nd international conference on Machine learning - ICML ’05. Bonn, Germany: ACM Press, 2005: 625–32.

17 Efron B, Tibshirani RJ. An introduction to the bootstrap. CRC press, 1994.

18 Lundberg SM, Lee S-I. A Unified Approach to Interpreting Model Predictions. *Adv Neural Inf Process Syst* 2017; **30**: 4765–74.

**Legends for figures and tables for supplementary appendix (in order of appearance):**

**Table A.**  Summary of included studies

**Figure A.** Data for 4,131 patients with columns indicating variables white lines representing missing data.

**Figure B.**  Pearson correlation grid for features included in the final models to examine for collinearity. Minimum, median platelets and haematocrit were correlated with a coefficient of 0.9 and 0.81 respectively but other features were generally independent from each other.

**Table B.** Univariate analyses between development and hold-out test set after random stratified splitting, comparisons made by Mann-Whitney testing.

**Figure C.** Summary plot for the variables used in the model in descending order of importance. Dengue outcomes are encoded as 1: complicated, 0: non-complicated. The colour gradients represent the value of the predictor – for example a lower minimum platelet count was associated with a positive impact on the model output in predicting a complicated dengue outcome.

**Figure D.** Scatter plots of transformed predictor values (x axis) and SHAP values (y axis). Outputs from the XGBoost is displayed on the left column, and logistic regression on the right. SHAP values are an indicator of feature importance with regards to the model output for the model when tested against the hold-out set.

**Figure E.** Scatter plots of transformed predictor values (x axis) and SHAP values (y axis) across other models.

**Table C.** Clinical endpoints extracted from individual studies

**Table D.** Baseline description of patients (n=4,131) included in analysis. Haematological values and ranges refer to results taken over the initial 48 hours of hospitalisation only. Data is presented as median and brackets denote the interquartile range. Univariate analyses were done using the Mann-Whitney test.

**Table E.** Performance of models for each algorithm with respect to internal 10-fold cross validation on the development set (n=3,304). The 95% confidence intervals shown in brackets were derived from bootstrapping of the development set to 90% of original size and tested against the out of bag samples.

**Table F.** Performance metrics of final models when tested against the hold-out set (n=847).

**Table G** - Performance of XGBoost model in leave one group out cross validation

**Table H.** Summarised results for all models in leave one group out cross validation

**Table I.** Model performances in 10-fold cross valuation between studies using 1997 and 2009 WHO dengue treatment guideline classification.

**Table J.** Optimal performance metrics of different models in 10-fold cross validation using the paediatric subset only (n=3,571)

**Table K.** Test performance of the models trained on the paediatric cohort and applied to the adult set.
