# Supplementary material for: Applied machine learning for the risk-stratification and clinical decision support of hospitalised patients with dengue in Vietnam
Source: PLOS Digit Health. 2022 Jan 18;1(1):e0000005. doi: 10.1371/journal.pdig.0000005 (PMC9931311; doi:10.1371/journal.pdig.0000005)
Supplement: Attachment — Submitted filename: Response to Reviewers.docx [file pdig.0000005.s002.docx]

Journal Requirements:

1. Please provide separate figure files in .tif or .eps format only, and remove any figures embedded in your manuscript file. If you are using LaTeX, you do not need to remove embedded figures.

Thank you – figures removed from manuscript and tif images provided.

2. Please amend your detailed Financial Disclosure statement. This is published with the article, therefore should be completed in full sentences and contain the exact wording you wish to be published.

i). Please include all sources of funding (financial or material support) for your study. List the grants (with grant number) or organizations (with url) that supported your study, including funding received from your institution.

Funding and code has been added to financial disclosure statement

ii). State the initials, alongside each funding source, of each author to receive each grant.

N/A

iii). State what role the funders took in the study. If the funders had no role in your study, please state: “The funders had no role in study design, data collection and analysis, decision to publish, or preparation of the manuscript.”

This statement has been moved from line 222 to the financial disclosure section

iv). If any authors received a salary from any of your funders, please state which authors and which funders.

Added

3. Please update the completed 'Competing Interests' statement, including any COIs declared by your co-authors. If you have no competing interests to declare, please state "The authors have declared that no competing interests exist". Otherwise please declare all competing interests beginning with the statement "I have read the journal's policy and the authors of this manuscript have the following competing interests:

Added.

4. In the online submission form, you indicated that "The original clinical datasets used contains identifying or sensitive patient information relating to past and ongoing clinical studies and access is subject to research ethics committee approval from HTD ethical committee and OxTREC approval. Please contact the authors for further information and data requests.". All PLOS journals now require all data underlying the findings described in their manuscript to be freely available to other researchers, either 1. In a public repository, 2. Within the manuscript itself, or 3. Uploaded as supplementary information.

Thank you - Permissions and processes for an anonymised version the dataset used for the analysis is currently being uploaded to the public repository hosted by Oxford University (<https://ora.ox.ac.uk/>). We expect that the process will be complete in the upcoming weeks and the data availability statement can be updated at time of potential publication to directly link to the dataset used.

To the reviewers:

We are very grateful for the time taken for your comments on the manuscript. These have been addressed point-by-point below in blue, with corresponding changes tracked in the attached manuscript. Please do not hesitate to contact us if we can provide additional clarification.

Yours sincerely,

Damien Ming

Sophie Yacoub

On behalf of the authors and the VITAL consortium

---

**Reviewer #1:**

The paper presents the use of machine learning algorithms to predict dengue shock syndrome based on an integrated dataset originating from 5 different studies. Overall, the research methodology and the methods are well presented. The issues with the work are as follows:

(i) The selection of the predictors is opportunistic and hence it is unclear whether there are other clinical features that should have been included. The list of predictors is quite simplistic from a clinical perspective, though the positive is that these predictors still are given high prediction accuracy;

Thank you – we agree that features included in the final model (day of illness, age, sex, weight and indices of haematocrit and platelets in the first 48 hours of hospital admission) are relatively basic in nature. The strengths of these features are that that their measurements are objective and uniform across studies, and there was minimal missing data. Our work serves as an initial exemplar to illustrate the role of machine learning in supporting clinical decision-making within dengue, which has not been previously demonstrated.

We are conscious that in developing models for LMIC settings such as Vietnam, the feasibility of implementation is crucial. Therefore, possible features used in these models should be limited to those which can be routinely accessed in these settings. However, we do plan to build on the model through incorporation of more complex diverse data: one strand of our project involves collection of physiological signals in the form of photoplethysmography using low-cost, scalable wearables (vital.oucru.org). This will hopefully allow us to improve the strength of our predictions.

(ii) Given the limited number of predictors, there is no opportunity to perform feature selection which is a limitation;

We agree. The availability of additional and diverse features could lead to novel insights and exploit strengths of machine learning from a Big Data perspective. A higher dimensional dataset would allow for feature selection and/or engineering. We emphasise this limitation in the discussion (lines 378).

However, datasets from clinical trials examining severe dengue were utilised for model development, feature selection processes have already taken place at the level of study design. The features used in our study are known to be significant for dengue risk prediction and are in accordance with WHO 2009 dengue treatment guidelines, which widens their applicability across healthcare settings.

(iii)	Correlation between the features and the outcome should be checked, and confounders should be established;

Thank you – we have examined for collinearity between features using Pearson’s correlation. Please see the supplementary appendix page 9 for full results. We find that each of the features included in the model generally do not correlate with each other but the median platelet and minimum platelet count do so (coefficient of 0.9). The fall in platelets during early illness in the few days during the febrile phase is well-described in dengue and is likely to account for these.

(iv)	The prediction models perform binary classification, where there is a significant class imbalance such that only 5.4% of patients had a positive dengue shock. The study does not use any methods to address the class imbalance, and it is interesting to note such a high sensitivity reported in the results despite the class imbalance. Furthermore, which such a low number of the dengue class the authors should point out the ratio of the two classes in both the training and evaluation sets. the study can be improved by performing data augmentation using GAN or simply using SMOTE and then compare results between the original and the augmented dataset;

The class imbalance in our dataset with 5.4% experiencing shock is typical of hospitalised patients and commensurate with observed real-world settings and data. The ratio of the two classes in the training and evaluation set is the same (5.4% and 5.3% respectively) as a stratified split was used – we now have made this clearer in the manuscript (line 186).

We have conducted additional analyses by incorporating SMOTE into our grid search pipeline. We did not find significant differences between the results in terms of AUROC for our models in cross-validation and hold-out testing, apart from SVC which improved the overall score (AUROC from 0.770 to 0.819). Thank you for these suggestions, we updated these new results and repeated the bootstrapping process and this is displayed in the amended manuscript.

(v)	Table 1: is there an explanation for why the dengue shock numbers are so high in cohort S1 despite cohort S4 has a higher number of patients. It seems that there may be some issues with the inclusion criteria that need proper investigation;

Cohort S1 recruited children already hospitalised during the febrile phase and cohort S4 recruited children from the community, who were subsequently hospitalised. One possibility is that patients in S4 who developed severe disease may not have been hospitalised locally (to our study hospitals) or were lost to follow up resulting in a difference in rates of severe dengue.

Patients in these cohorts (S1-S5) were included in our study as they: have a definite diagnosis of dengue, are admitted to hospital in Ho Chi Minh City, have the requisite data available and have not already developed shock during this period. The outcomes used are also standardised across studies (See methods).

We acknowledge differences exist between the cohorts. Each cohort is different as the studies themselves are based on different research questions. Patients included may also be subjected to different inclusion criteria and clinical triage processes (see below), access to healthcare may also be different depending study. Furthermore, seasonality during the different recruitment periods will influence the dengue caseload, with studies conducted during seasonal epidemics or during circulation of particular virus serotypes resulting in more severe disease seen.

Finally, during the data processing step we excluded patients who were already admitted with, or developed severe dengue within the 48 hours of admission. This rate varies across different studies and further contribute to differences in the rates of dengue shock between cohorts.

(vi)	The aggregation of data from multiple temporal and institutional cohorts should take into account the clinical interpretation of dengue shock across these institutions to have a standardized inclusion criteria. It would be useful to develop independent models for each cohort to investigate the influence of the different predictors across cohorts;

We performed additional sensitivity analyses to examine the impact of these cohorts on performance by looking at the individual studies and by separating the dataset depending on the WHO definitions employed (1997 vs 2009 guidelines).

Using optimised models, we used a leave one group out approach for each study using the training dataset. We found variations in AUROC in 10-fold cross-validation: for the XGBoost model median AUROC was 0.78 (IQR 0.50 to 0.83) however for the logistic regression model the median AUROC was 0.84 (IQR 0.80 to 0.90). Limitations are that these cohorts are of considerably different sizes (47 to 2,615 patients) and will contribute to this variance in performance.

When the whole dataset is split according to the 1997 or 2009 WHO definitions employed, there also exists differences in optimal model performance although it is noted that studies which employ the 2009 WHO definitions (S2-S4) are much more heterogenous in their design.

This is a limitation to our work and we have expanded on this in lines 353 of the discussion and in the supplementary appendix pages 19-20.

(vii)	The data spans 19 years which is a rather long period given improvements in diagnostic methods and treatments--as the authors suggest that this study points to the utility of the model in LMIC given the need for a limited number of clinical attributes, it will be useful to stratify the data into temporal periods and then develop prediction models for each period to confirm that the predictors hold there importance across time;

We agree that developments have occurred over such a period, but specifically for dengue we would argue that this has mainly resulted in improvements on the management of the critically ill, which occurs after onset of dengue shock.

The clinical approach in dengue diagnosis and risk prediction have not varied significantly over time, and thus forms a major motivation for our current study. Performance of diagnostics used (namely RT-PCR and NS1 from blood) also remain similar over time. Treatment for dengue is fully supportive – and although intravenous fluid regimens may have changed - these effects would not apply to the febrile, pre-shock states which is the focus of this work (i.e. medical treatment would not have a significant impact on the patients who would ultimately develop dengue shock).

Nonetheless a temporal window approach to evaluate changes over time would be of major interest but we feel beyond the scope of the current study.

(viii)	The data does not distinguish between paediatric patients and adults/elderly patients despite both groups have different clinical characteristics, especially elderly having co-morbidities. Therefore, having a universal model for all age groups is susceptible to bias towards a specific age group.

Thank you – this is an important point and we have performed additional sensitivity analyses to address these. We show that using a paediatric cohort only (defined as younger than 18 years of age, n=3,571) robust model performances are possible: median cross-validation AUROCs from all models around 0.82 (IQR 0.810 to 0.823) can be attained. When these models are applied to adults, performance reduced although there exists a major class imbalance in this adult test set (n=499, 13 cases of dengue shock, 2.6%). Future iterations of the model will need to balance overall generalisability against optimisation for specific age groups and we include this in the discussion lines 382. Please see supplementary appendix page 20 for results.

(ix)	The authors should provide details of how the trained models were calibrated;

We used isotonic calibration with 10-fold cross-validation to calibrate the trained models. This has been clarified in the methods section (lines 211)

(x)	The discussion should include insights into the clinical pragmatics of this work, the actual design and implementation of the digital health based solution in a clinical workflow, alluding to data access interfaces, etc. Also, the clinical setting in which it will be used and how will its use be determined on a per patient basis;

Thank you for this very important comment. The digital health solution is based on a web interface designed by one of our co-authors <https://doi.org/10.25560/73000> (BH) and can be displayed on a standard browser. The flow of the system is vital to usability and in parallel we are conducting implementation studies examining the workflow, end-user needs and optimal design of the interface. We are acutely conscious that the machine learning module should only form part of the functionality of a clinical decision support system. We have elaborated on this in the discussion and plans for implementation in lines 311 of the discussion.

(xi)	The authors should comment on the scalability of this model to other regions;

This is the subject of investigation by one of our co-authors (SS) <https://doi.org/10.1016/j.ijid.2020.11.056> . It has been shown that machine learning models trained using data from one region (Vietnam) can feasibly be applied to another setting (Thailand) with reasonable performance. Limitations which have been highlighted from this work is that although the disease phenotype and classification systems may be similar, differences in the healthcare processes contribute to significance variance between datasets. Additional factors such as disease endemicity, access to healthcare services also need to be addressed in order for systems to be scalable. This has been expanded in our discussion in lines 314.

(xii)	What plans are in place to keep the model current given the availability of new data and changes in clinical processes;

We agree that continuous review and improvement of the model is essential to ensure that performance remains clinically relevant. We plan to validate the models prospectively in real-world clinical settings as a next step; patients are being recruited into these studies although COVID-19 has caused significant delays. New data especially PPG signals are planned to be incorporated into the decision support system in order to allow for more individualised predictions.

**Reviewer #2:**

The authors have ~4000 patients from 5 different data sources, and are trying to make different models (XGBoost, Logistic Regression, ANN, Random forest and SVM) to classify the patients according to if they have DSS (Dengue shock syndrome) they also do a secondary analysis making models for complications in DSS they have also done SHAP interpretability analysis for xgboost and logistic regression. Overall, the paper was well written we have some points that we found could be beneficial to improve the quality of the work

Major comments:

i) Authors should mention the effect of different data sources, i.e. data batches. Specifically, if the sampling strategy employed addresses potential biases due to discontinuous data collection in batches over the years. technologies/approaches must have changed over the long period of collection. It is important to account for these batch effects as they may dramatically change the outcome.

Thank you for this very important point.

Differences do exist between data sources used. These inherent differences arise from the different research questions posed by each clinical study. For example, the population in studies S1, S2 and S4 are predominantly children whereas S3 and S5 included only adults. Study S1 recruited patients directly from the hospital but S4 patients from the community. Other factors including seasonality, different recruitment sites, changes in clinical methods play a role in influencing the patient inclusion process. This almost certainly will result in a patient cohort which is not reflective of what might be routinely encountered in real-world settings.

We feel that the inclusion of these different studies as one of the strengths of this study. Patients within each batch are sufficiently similar to each other (they are all admitted to hospital with a confirmed diagnosis of dengue, clinical data collected during the febrile phase and prior to development of dengue shock) to be comparable and included in a single analysis. But they also importantly provide the variance needed to train models which are likely to perform well prospectively.

We believe from clinical experience that developments in dengue over the period of 19 years have focused mainly on the management of patients who have *already* developed dengue shock but the clinical approach to the patient assessment has remained relatively unchanged (hence the motivation for our study).

However, we fully agree with you that there will be batch effects as a result of aggregating different data sources of different sizes over a long period of time. These are multiple, complex and we feel beyond the scope of our current approach (see below). For these reasons we have not explicitly corrected for batch effects through individual normalisation or other batch correction algorithms, in order to maintain these subgroup effects in the final model.

There are definite strengths and weaknesses to this assumption and we expand on this in lines 353 of the discussion, in supplementary appendix page 19, and also addressed in the reply to the previous reviewer (points v – vii).

ii) As a suggestion, to reduce the bias in the model caused by data coming from 5 sources, and subsequently increase its generalization performance, the authors could have normalized the data from each source separately and then concatenated these 5 normalized datasets into one, instead of first concatenating and then normalizing. The authors haven’t mentioned when they performed the normalizing step but since they mentioned it at the end in line 177, it is reasonable to assume that it was the last step. Having clarity on how this normalization was performed and at which step during the process is important.

Thank you – pre-processing (namely imputation of the ~ 1.5% missing values and scaling normalisation) was done in our study after concatenation using data from the training set only.

We feel that the batches vary too significantly with respect to certain features that normalisation (such as scaling) before concatenation would not produce satisfactory results. For example, the median age in S1 is 12 years whereas the median age in S5 is 30 years old. Patient age differences will also have subsequent effects on their weight, and normal population distributions of haematocrit and platelets.

We have been more explicit on this matter again in the discussion (lines 353), with the assumption that the features across patients in different batches are sufficiently comparable to provide meaningful results as per the reply above – however again acknowledging that there are no straightforward ways to account for all this.

iii) Also, the entire analysis and the deidentified data should be hosted on a public platform such as github to allow researchers to reproduce the work.

Thank you – the deidentified dataset is currently being uploaded and made available to public access through the Oxford University Research Archive online repository (ORA). This will be made available and accompany the manuscript at the time of potential publication. The code used to conduct these analyses will also be provided to the journal and uploaded for access.

Minor comments:

1) In Abstract Findings : The authors should give the distribution of patients who experienced DSS (how many of them were adults and how many were children)

This has been added in line 231: Out of the 222 patients who developed DSS 13 (5.9%) were adults and 209 (94.1%) were children.

2) Line 146: The authors should mention what symptoms they have taken to be ‘onset of illness’ in patients who did not develop DSS.

This has been added in line 147. The onset of fever was taken as the measure of illness onset (time zero) given that fever is almost universal and is used as a measure in many dengue studies.

3) Line 177: Predictors used in the model which were transformed should be mentioned.

Thank you – this has been added in line 178.

The authors could specify the Brier scores for evaluation against the hold out set for the primary analysis too, as they have done for the secondary analysis (Supplementary Appendix 3)

Apologies this was omitted. We have added these now into the table 4.

Supplementary Appendix 2: In the hyperparameters of the XGBoost model, the authors mention 2 optimal models. The authors should specify if these have equal AUROC, or if one was chosen because of less overfitting and other for better performance, or whatever else the case might be. Also, the ‘min_child_weight’ in both of these is different from the set of values they have taken initially (0.005 and 0.001 are not present in [0.05, 0.1, 0.2])

Again, many apologies and thank you for pointing this out – this was a duplication error and we used a single final model after the grid search which has now been amended in the supplementary appendix page 10. We also performed an extended XGBoost grid search (see comment below) but were not able to improve on the performance.

Supplementary Appendix 2: As a side note, the authors have taken very few parameters into account for the XGBoost model, and it could be possible to achieve a better AUROC using hyperparameters like ‘subsample’, ‘colsample_by...’ and ‘alpha’ and using a more extensive set of values for parameters like ‘max_depth’ and especially ‘min_child_weight’, for which the values used are quite small in magnitude.

Thank you for the suggestions –grid search parameters have been extended for XGBoost model looking at greater ‘eta’, ‘min_child_weight’ and ‘max_depths’ values, alongside different values for the ‘subsample’, ‘cosample_by_tree’ and ‘alpha’ parameters from their defaults (1, 1 and 0 respectively) with AUROC as the scoring metric on the development set. We found that the optimal score achieved in 10-fold cross validation was 0.81 which was the same as the other model. Given the relatively limited set of predictors and consistent performance of the different models we feel that this discrimination is close to one which could be achieved by an optimal model.

Grid search space:

'eta': [0.05,0.1,0.2],

'min_child_weight': [0.05,0.1,0.2],

'max_depth': [5,7,10],

'gamma': [0.05,0.1,0.2],

'n_estimators':[25,100,200],

'subsample': [0.25,0.5,1],

'cosample_by_tree': [0.5, 0.7, 1],

'alpha': [0, 0.2, 0.5]

Supplementary Appendix 2 (Interpretability and SHAP analysis): SHAP is only applied for XGBoost and logistic regression. The authors can use SHAP’s KernelExplainer to explain any model. However, making the MLP(ANN) using PyTorch/Tensorflow/keras and then using SHAP’s DeepExplainer or GradientExplainer classes will lead to better explanations and interpretability.

SHAP was applied for XGBoost and logistic regression to illustrate the contrasting algorithms (tree based versus linear). We have taken your suggestion to perform SHAP analyses for all optimised models in our study.

We found that a lower minimum platelet count and higher maximum haematocrit tended to be favoured across the models as a predictor for dengue shock. Interestingly a lower minimum haematocrit was also associated with dengue shock classification suggesting the magnitude and dynamics of haematocrit change over this period may also have predictive value. This can be seen clinically in dramatic fluid shifts as a result of leakage as well as intravenous fluid replacement during resuscitation. Please see supplementary appendix 14-15 for these additional summary plots.

Reviewer #3:

It was a delight to review this paper, which in its present form I could readily imagine appearing in the journal. I only have a few comments for the authors to address.

L174: Provide more information on how imputation was done. I can’t work out from this description how the mean or median was selected.

Apologies for the ambiguity – both mean and median values were imputed as part of a grid search process. Whichever method provided the better performing model based on the AUROC in the cross-validation was then chosen and taken forward for hold-out set testing. This has now been elaborated more fully in lines 174.

L176: Please provide a better explanation of which data were standardised and how the decision was made to standardise. Don’t some of the algorithms (such as lasso) automatically standardise covariates?

Thank you – yes for certain algorithms such as random forest standardisation makes little difference but for others such as neural network they do. Like for imputation, we used an empirical grid search process in order to see if either standardisation or no standardisation provided the better performing model for each algorithm (lines 178).

L202: I was surprised to see the optimal cut off being determined through a statistical algorithm rather than through the clinical requirements. I was expecting the decision to be based on, for instance, a maximum tolerable number of patients with DSS being sent home (presumably to die) or the hospital capacity, rather than Youden’s J statistic.

Thank you for this important point. We used the J statistic in order to maximise specificity and sensitivity in order to provide a good all-round performance.

But we do agree that maintain specific metrics above a threshold, such as ensuring that the negative predictive value is clinically relevant (and optimal for the clinical setting) would be a good strategy. Further work is currently ongoing to understand what the best role for this model is (i.e. as a rule in or rule out test) and how best to implement these. We have expanded on this in lines 331 of the discussion.

L237: Was stochastic domination assured for these p-values?

The p-values calculated for Table 2 were univariate comparisons using the Mann-Whitney or Chi squared tests between those who developed shock or not in their hospital stay. This was done on the dataset aggregated from studies 1-5. We are unclear on the application of stochastic domination for this but would be happy to clarify our methods further if this is helpful.

L260: What is linear logistic regression?

This has been amended to “Logistic regression” only.

L269 (figure 2): This is very pretty but probably requires a bit more information to guide readers’ interpretation. For instance, with sex, how do we interpret the different shades of blue and red and white? Also I wonder whether the plot should be taller so that the densities are easier to perceive.

Thank you – we have added more text to the caption of the figure to assist with interpretation. A larger version of the figure has been submitted to the editors for reproduction and the densities will hopefully now be clearer to the reader.

The intention of the figure is to illustrate extremes of feature values in certain cases contribute towards the classification (e.g. high maximum haematocrit, and low minimum platelet count contribute towards shock). Female sex is encoded as 0 (blue) and male sex encoded as 1 (red) but we agree with you that the mixture of densities can be difficult to interpret by themselves and is a limitation of this plot.

L316: Please check and confirm that the Tan Tock Seng Hospital (Singapore) dengue clinical cohort is not larger. I had the impression it was similar in size, and they’ve used it for similar applications, though obviously their patients are quite different.

Thank you – the group does excellent work and most recently they developed a dynamic model to predict onset of dengue haemorrhagic fever (DHF) for hospitalised patients, using a cohort of 1,771 individuals (<https://doi.org/10.1093/trstmh/trz099>). However the patients included were also all adults, and dengue shock (i.e. the most severe classification of DHF) was not used as an endpoint.

Data availability: It would be preferable if a sanitised version of the dataset could be provided, with just the variables that were included in the final analysis so no need for information that could identify the patients.

The deidentified dataset is currently being uploaded and made available to public access through the Oxford University Research Archive online repository (ORA). Access will be available at the time of potential publication. The code used to conduct these analyses will also be provided to the journal and uploaded for access.
